# Perspectives on Self-Management and Digital Tools for Individuals With Type 2 Diabetes in Flanders: Qualitative Focus Group Study

**DOI:** 10.2196/88446

**Published:** 2026-10-27

**Authors:** Barbara Annancy, Pieter Van Bostraeten, Charlien Janssen, Steve Van den Bulck, Roman Vangoitsenhoven, Geert Goderis

**Affiliations:** 1 Academic Center for General Practice, Department of Public Health and Primary Care, Faculty of Medicine, KU Leuven, Kapucijnenvoer 7 Blok G-Bus 7001, Leuven, Flanders, Flemish Brabant, Belgium, 32 16330078; 2 Endocrinology, Department of Endocrinology, University Hospital of Leuven, Leuven, Flanders, Flemish Brabant, Belgium; 3 Research Group Healthcare and Ethics, Department of Care & Ethics, Faculty of Medicine and Life Sciences, Hasselt University, Hasselt, Flanders, Limburg, Belgium; 4 Department of Chronic diseases and Metabolism, Clinical and Experimental Endocrinology, KU Leuven, Leuven, Flanders, Flemish Brabant, Belgium

**Keywords:** type 2 diabetes mellitus, diabetes self-management, digital health, patient education, multidisciplinary care, qualitative research, thematic analysis, focus group discussion, Flanders

## Abstract

**Background:**

Type 2 diabetes (T2DM) is one of the leading chronic diseases worldwide, requiring effective long-term self-management (SM) to improve health outcomes and reduce complications. Although structured diabetes pathways exist in Belgium, national reports indicate that adherence to the recommended diabetes follow-up and routine monitoring remains suboptimal. Digital health technologies have shown promise in supporting SM; however, little is known about how stakeholders within the Flemish health care system perceive opportunities to improve SM and implement digital health interventions.

**Objective:**

This study aimed to explore stakeholders’ perspectives on improving diabetes SM and the potential role of digital tools in supporting people with T2DM in the Flemish health care system.

**Methods:**

A qualitative study was conducted using 2 online semistructured focus group discussions with 19 stakeholders, including health care professionals (HCPs) from primary, secondary, and tertiary care settings; researchers; digital health experts; and an individual living with T2DM. Participants were recruited using maximum variation purposive sampling followed by snowball sampling. The discussion guide was informed by the Consolidated Framework for Implementation Research and current evidence on diabetes SM and digital health. Discussions were audio-recorded, transcribed verbatim, translated from Dutch into English, analyzed using Braun and Clarke’s 6-phase reflexive thematic analysis, and supported by NVivo (QSR International).

**Results:**

Five key themes were identified: (1) Empowerment through education, (2) Tailoring education to individual needs, (3) Multidisciplinary and cross-sectoral collaboration, (4) Opportunities for digital technology in self-management, and (5) Regional and national systemic limitations. Participants consistently emphasized that effective diabetes SM may extend beyond patients’ individual behaviors and depends on tailored education, coordinated multidisciplinary care across the health care sectors, supportive organizational structures, and supportive health policies. Digital health technologies were perceived as valuable tools to facilitate patient education, communication, information sharing, and continuity of care. However, participants also highlighted that successful implementation depends on addressing broader organizational and system-level barriers, including fragmented digital infrastructures, limited interoperability, and regulatory constraints within the Belgian health care system.

**Conclusions:**

Stakeholders viewed improving diabetes SM as a shared responsibility between patients, HCPs, and the health care system. While digital health technologies may strengthen SM support, their value depends on how well they complement existing care processes and address contextual implementation challenges. These findings provide context-specific insights that may inform the development and implementation of digital interventions for T2DM in Belgium and similar health care settings. Further qualitative research involving a larger and more diverse sample of people living with T2DM is warranted to better understand patient experiences and support needs.

## Introduction

Diabetes remains one of the most prevalent noncommunicable chronic diseases, with the World Health Organization reporting that the disease was directly responsible for 1.6 million deaths in 2021 and that an estimated 830 million people worldwide were living with diabetes. Predictions estimate that 1.3 billion adults will be diagnosed with this disease by 2050 [1,2]. Type 2 diabetes mellitus (T2DM) accounts for 90%‐95% of diabetes cases, underscoring its predominance and the urgent need for sustainable and scalable management strategies. In Belgium, approximately 7.6% of the population received a formal diagnosis of diabetes in 2023 [3]. However, national reports estimate that 1 in 3 Belgians with diabetes remains undiagnosed, suggesting a true prevalence of more than 10% [3]. Beyond its increasing prevalence, T2DM imposes a significant burden through long-term microvascular and macrovascular complications that contribute to morbidity, disability, and premature mortality [4].

The 2022 consensus report by the European Association for the Study of Diabetes and the American Diabetes Association emphasizes the importance of combining pharmacological therapy with multifactorial management [5]. Key elements for optimal diabetes management and self-care activities include a person-centered approach, structured diabetes self-management (SM) education and support, behavioral strategies (including regular physical activity and smoking cessation), and the active involvement of individuals in treatment decisions and during therapy goal setting. Accordingly, there has been a shift from clinician-led care toward collaborative models that allow engagement and SM. Structured SM support has consistently been associated with improved metabolic control, self-care behaviors, other clinical outcomes, and reduced diabetes-related complications [6-8].

Belgium’s health care system is recognized for its accessibility and high-quality service delivery [9]. For T2DM care, 3 structured pathways have been established: the diabetes start trajectory, the diabetes care trajectory, and the convention pathway, each tailored to patients’ needs and levels of disease complexity [10,11]. The pathways are organized as contracts that state minimal requirements, in which all partners agree to be actively involved, and the administrative burden is minimized. The start trajectory primarily targets patients managed in primary care with lifestyle interventions and/or oral medication. The care trajectory encompasses patients who require up to 2 daily injections of insulin or other glucose-lowering drugs (eg, GLP1-receptor agonists). These patients are followed primarily at the office of their general practitioner (GP) and are required to see an endocrinologist only once per year. Individuals with complex insulin regimens involving at least 3 injections per day or continuous subcutaneous insulin infusion are assigned to the convention pathway, where the main follow-up is organized in specialist endocrinology centers (with ≥2 visits per year). These pathways extend across both primary and specialized care sectors. Depending on the pathway, patients are required to engage in regular consultations with GPs and diabetologists, as well as structured formal education sessions with diabetes educators, dietitians, and podiatrists. Additionally, patients typically benefit from substantial reimbursements and access to diabetes technologies (such as continuous glucose monitoring) at a subsidized rate [12,13].

Despite these well-established care pathways, important gaps remain in the delivery of recommended diabetes care. The 2024 Belgian Health System Report by Gerkens et al [9] demonstrated that the proportion of individuals receiving the recommended diabetes follow-up remains suboptimal across several age groups. In this context, *recommended diabetes follow-up* refers to the timely completion of the minimum Belgian guideline–recommended monitoring and routine clinical procedures, including biannual hemoglobin A_1c_ (HbA_1c_) measurements, annual albuminuria assessments, serum creatinine and lipid measurements, retinal screening, and foot examinations [14,15]. Similarly, De Baets et al [13] identified persistent gaps in the optimal management of people living with T2DM in Belgium, underscoring the need for systemic improvements. Although these challenges appear multifactorial, effective diabetes management depends not only on the availability of health care services but also on patients’ ability to engage in recommended SM behaviors, including monitoring their condition, adhering to treatment recommendations, and communicating with health care professionals (HCPs). Improving SM may therefore represent one potential strategy for improving adherence to recommended diabetes care processes [13].

Advances in information and communication technology (ICT) with several digital systems, such as mobile health apps, patient portals, and digital medical platforms, have introduced opportunities to enhance health management, improve care quality, reduce medical errors, and facilitate communication between HCPs [16-18]. Several randomized controlled trials have reported significant improvements in glycemic control through interventions supporting blood glucose self-monitoring, real-time feedback, remote consultations, and behavioral support [19-21].

Although numerous studies and systematic reviews have explored diabetes SM and digital health interventions internationally, health care systems differ substantially in their organization, reimbursement strategies, digital advancements, and multidisciplinary care pathways [22,23]. Furthermore, successful implementation of these interventions is highly associated with the organizational, professional, technological, and policy context in which they are introduced [24,25]. Consequently, findings from other countries cannot be assumed to be directly transferable to the Belgian health care system.

To our knowledge, few qualitative studies have explored how diverse stakeholders within the Flemish health care context perceive opportunities to improve diabetes SM and how digital technologies could be implemented to support these practices. Therefore, this qualitative study aimed to explore stakeholders’ perspectives on strategies to enhance SM practices and to examine the potential role of health ICT in supporting these processes. By providing insights into the perspectives of HCPs, researchers, digital health stakeholders, and individuals living with T2DM, this study contributes context-specific evidence that may inform the development and implementation of approaches to strengthen diabetes SM within the Flemish health care system, with potential relevance to other health care settings facing similar challenges.

This qualitative study conducted within the Digital Diabetes Patient Reminder (DIPAR) [26] pilot in Leuven, Belgium, one of the 143 pilot interventions of the Joint Action on Cardiovascular Diseases and Diabetes (JACARDI). JACARDI is a recent EU4Health Program that aims to reduce the burden of cardiovascular disease and diabetes by uniting 21 European countries that aim to target and address every aspect of the patient journey [27].

The DIPAR pilot aims to develop, implement, and evaluate the DIPAR tool, which has the potential to transform diabetes care by enhancing both clinical and process outcomes through a mixed methods analysis. This pilot is assigned to a work package within JACARDI for SM that focuses on various aspects of patients’ SM, including making lifestyle changes, self-monitoring symptoms and self-treatment, communicating with care professionals, and coping with the consequences of the disease and treatment in daily life [26-28].

## Methods

### Design and Setting

This study complied with the COREQ (Consolidated Criteria for Reporting Qualitative Research) [29] and can be found in Checklist 1. A qualitative study design using focus group discussions was used to explore the perspectives of key stakeholders on how to improve SM practices for individuals with T2DM and the potential role of implementing digital tools in Flanders, Belgium. Focus groups are known to enable dynamic interaction and facilitate the identification of individual and shared views regarding the topics, and therefore this form of qualitative research was selected.

### Ethical Considerations

This study obtained ethical approval from the research ethics committee at UZ/KU Leuven (internal reference number: S68985). Written informed consent was obtained prior to study participation and data collection, including consent for audio and video recording (Multimedia Appendix 1). Verbal confirmation of consent was also obtained at the beginning of each focus group immediately before recording commenced. Participants’ data were pseudonymized, and video and audio recordings and pseudonymized transcripts were stored securely on KU Leuven servers in accordance with the General Data Protection Regulation and Belgian data protection regulations. No financial incentive or other compensation was provided. This study was conducted in accordance with the principles of the Declaration of Helsinki [30].

### Participants and Recruitment Strategy

The recruitment strategy aimed to include a multidisciplinary sample of HCPs, researchers, digital health experts, and individuals with lived experience of T2DM to capture a broad range of perspectives on diabetes SM and digital health implementation. Eligible HCPs included professionals directly involved in the treatment and care of people with T2DM, as well as those indirectly supporting diabetes management across primary, secondary, and tertiary health care settings in Flanders. Individuals living with T2DM who were receiving diabetes care were also eligible to participate.

Participants were recruited using purposive sampling through the research team’s professional networks, followed by snowball sampling to identify additional information-rich participants with relevant expertise [31]. Invitations were distributed by email and included information on the study objectives, design, and procedures. Participant recruitment was initially intended to be facilitated through a regional patient association. However, this recruitment pathway could not be implemented as no timely response was received due to an internal restructuring within the association. Consequently, individuals with T2DM were recruited through their established therapeutic relationships with participating GPs and endocrinologists. To facilitate participation from stakeholders across Flanders and maximize professional and geographical diversity, the focus groups were conducted online in accordance with best-practice recommendations for virtual qualitative research by Richard et al [32].

Participants were allocated to 1 of 2 focus groups based primarily on their availability while ensuring that each group included a diverse mix of HCPs, researchers, digital health experts, and stakeholder perspectives. This heterogeneous composition was intentional, as the study sought to stimulate discussion across professional boundaries and explore how different stakeholders perceived opportunities to improve diabetes SM and implement digital health interventions.

### Eligibility Criteria

Eligible participants included GPs, diabetes educators, nurses, dietitians, pharmacists, hospital-based diabetes experts and endocrinologists, software and ICT providers or developers, and researchers in the field of public health, chronic diseases, and diabetes, all of whom were actively involved in the care of individuals with T2DM or supported the management of diabetes. Participants with lived experience were required to have a confirmed diagnosis of T2DM and to be under the care of a GP or endocrinologist. Minors, hospitalized individuals, or people with cognitive impairments were excluded. All participants were required to be fluent in Dutch or English.

The maximum variation purposive sampling as described by Nyimbili and Nyimbili [33] was used to allow diversity in gender, professional role, and years of experience. Each focus group included about 9‐10 participants. The literature shows that focus groups of 6‐10 participants are commonly recommended for heterogeneous stakeholder groups to facilitate interaction while ensuring representation of diverse perspectives [34-37]. Given the multidisciplinary nature of the study, the selected group size enabled meaningful discussion and interaction among the stakeholders from different health care sectors and professional backgrounds while ensuring broad representation of relevant expertise. Written informed consent was obtained prior to study participation and data collection. No financial incentives were provided.

### Discussion Guide Development

A semistructured focus group discussion guide was developed to facilitate open discussion while ensuring that topics relevant to the study objectives were consistently explored across the focus groups. The guide was informed by the Consolidated Framework for Implementation Research (CFIR) [25], which served as a conceptual framework during the study and guide design to identify implementation determinants relevant to improving SM support and potential implementation of digital health interventions within the Flemish health care context. Additionally, the guide was informed by published literature on diabetes SM and digital health interventions, together with evidence identified through an ongoing systematic review conducted by the research team and expertise from members of the research team.

The initial guide was developed by 2 members of the research team (BA and GG) and subsequently reviewed and refined by the wider multidisciplinary research team through iterative discussions until consensus was reached. This process ensured that the discussion topics aligned with current evidence, the study objectives, and the Flemish health care context. Although the guide was not formally piloted, the same semistructured guide was used across both focus groups to ensure consistency in the topics explored, while allowing flexibility in the order and wording of questions to accommodate the natural progression of discussion and explore specific experiences or emerging topics in greater depth.

The guide comprised 4 sections: an introduction outlining the study objectives, discussion format, a warm-up with 1 introductory open-ended question and 2 prompts, 6 core open-ended questions with accompanying probes, and a concluding section. The core questions explored current SM support practices, potential barriers and facilitators to improving these practices in Flanders, the role of health ICT, and opportunities and challenges related to implementing digital tools for T2DM care. Participants were encouraged to freely share their perspectives and introduce additional topics relevant to the study objectives. Overall, the guide included 14 open-ended questions with accompanying subquestions or probes and allowed flexibility for participant-led discussion. The complete discussion guide is provided in Multimedia Appendix 2.

### Data Collection

Two focus group discussions of approximately 90 minutes were conducted in June 2024, after final recruitment, via the Microsoft Teams platform. To ensure thematic sufficiency in the study findings, participants who were unable to attend the scheduled sessions were also offered the opportunity to take part in individual interviews. No individual interviews took place.

The first focus group discussion was moderated by an experienced qualitative researcher (GG), GP and professor, and supported by a clinical endocrinologist and researcher (RV). The endocrinologist’s role was limited to assisting with moderation by monitoring discussion flow, clarifying clinical terminology when required, and ensuring that diabetes treatment and management in the specialized setting were appropriately understood and represented during the discussion. He did not contribute his own opinions or perspectives as a participant and was therefore not included as a discussant in the analysis. The second focus group discussion was moderated by the same senior researcher without assistance. Two team members (BA and CJ) observed both sessions.

Before participating in the discussions, participants were also provided with a short questionnaire (Multimedia Appendix 2) to collect demographic information. Each discussion began with a brief welcome and a short presentation outlining the objectives of the sessions and a brief background of the research team. No individuals other than the participants and members of the research team were present during the discussions, and no repeat focus group discussions were conducted.

### Data Coding and Analysis

All discussions were transcribed verbatim. Transcripts were pseudonymized and reviewed for completeness and accuracy against the original recordings. As the focus groups were conducted in Dutch, the transcripts were translated into English using DeepL Translate (DeepL SE). To ensure linguistic accuracy and preserve the intended meaning of participants’ responses, the translated transcripts were independently reviewed and compared with the original Dutch transcripts by 2 members of the research team: BA, a native English speaker with fluent proficiency in Dutch, and CJ, a native Dutch speaker. No discrepancies or ambiguities were identified during this process. The final English transcripts were imported into NVivo (version 14.23.0; QSR International) for data management and coding.

The data were analyzed using Braun and Clarke’s 6-phase reflexive thematic analysis [37-39]. An inductive reflexive approach was applied to identify patterns and generate insights from the data grounded in the accounts of study participants, rather than predefined theoretical and implementation constructs, in order to capture context-specific stakeholder perspectives relevant to diabetes SM and the potential role of digital tools in Flanders.

The 2 primary coders (BA and PVB) independently performed phrase-by-phrase coding of the transcripts in NVivo. Initial coding generated 100 and 122 unique codes from the first and second focus group discussions, respectively. The coders met after independently coding each transcript to compare interpretations and discuss similarities and differences. Coding was conducted in an iterative process, and similar codes were subsequently grouped into broader categories that reflected shared patterns across participants. Candidate themes were developed collaboratively through repeated discussions between the 2 coders and refined through several iterations by examining coherence within the themes and distinctions between the themes. Development of the thematic structure was continuously reviewed with the research team to ensure that the themes accurately reflected participants’ accounts and remained grounded in the data. Minor differences in interpretation occurred primarily regarding code allocation and theme boundaries. These were resolved through discussion and reexamination of the transcripts. Participant validation was not conducted, but final findings of this study were shared with all the participants.

### Trustworthiness

To increase trustworthiness of the study findings, several strategies that aligned with Lincoln and Guba’s criteria [40] were applied. Credibility was strengthened through investigator triangulation using independent coding by 2 researchers (BA and PVB) followed by reflexive discussions. The multidisciplinary focus group, bringing together stakeholders from different health care sectors and professional backgrounds, further enriched the discussions by facilitating the exchange of diverse perspectives and experiences. Transferability was supported through purposive sampling of stakeholders representing multiple professional disciplines and health care settings, relevant to the research topic, together with detailed reporting in accordance with the COREQ checklist. Dependability was supported through an iterative analytical process and documentation of coding decisions and theme development within NVivo. Confirmability was enhanced through discussions with the research team that challenged developing interpretations and ensured that the themes remained grounded in the data.

## Results

### Demographic Data

In total, 30 HCPs were invited to participate in this study, of whom 19 agreed to take part. The remaining invitees either did not respond to the initial email and follow-up reminders or were unavailable on the scheduled discussion dates. In addition, 4 individuals with T2DM were invited through their HCPs, but only 2 agreed to participate, both with prior experience of participation in qualitative research. Patient recruitment proved challenging despite sustained efforts.

Two focus group discussions were organized, each comprising 9‐10 participants. A total of 21 participants had agreed to participate in the focus group discussions; however, only 19 individuals ultimately took part, including 18 HCPs and 1 person with lived experience of T2DM. Two participants, 1 with lived experience and 1 psychologist, could not participate in the focus group discussion due to acute personal reasons.

As presented in Table 1, the majority of participants were female (11/18, 61.1%). Approximately 39% (7/18) were aged 51 years or older, while 27.8% (5/18) were aged between 31 and 40 years. More than 40% (8/18) of the professional sample comprised GPs and diabetes educators. The majority of discussion participants (14/18, >77%), including 2 digital health or ICT experts employed by a Belgian medical software provider, were primarily based in the primary care settings.

**Table 1.** Demographic characteristics of health care professionals in focus group discussions (N=18).

Demographic characteristics	Health care professionals
Health care professional role, n (%)	
Endocrinologist	2 (11.1)
General practitioner	4 (22.2)
Nursing staff member	1 (5.6)
Nursing diabetes educator	4 (22.2)
Dietitian	2 (11.1)
Pharmacist	1 (5.6)
Digital health (ICT)^a^ expert	2 (11.1)
Researcher	2 (11.1)
Experience in professional role (years), n (%)	
≤10	5 (27.8)
11‐20	4 (22.2)
21‐30	4 (22.2)
≥31	5 (27.8)
Professional setting, n (%)	
Primary	14 (77.8)
Secondary	1 (5.6)
Tertiary specialized	1 (5.6)
Research	2 (11.1)
Geographic location of professional role, n (%)	
Antwerp	4 (22.2)
East Flanders	2 (11.1)
Flemish Brabant	4 (22.2)
Limburg	4 (22.2)
West Flanders	2 (11.1)
Across Flanders (remote)	2 (11.1)
Age (years), n (%)	
25‐30	2 (11.1)
31‐40	5 (27.8)
41‐50	4 (22.2)
51‐65	7 (38.9)
Sex, n (%)	
Female	11 (61.1)
Male	7 (38.9)

a ICT: information and communication technology﻿.

Participants represented a broad spectrum of professional experience: approximately half reported 20 years or less experience in their respective fields, while the other half had more than 20 years of practice. In addition to the professionals, 1 participant with lived experience of T2DM participated in the first focus group discussion. This was a male individual in his late thirties and diagnosed approximately 3 years prior. This participant contributed their perspectives on living with T2DM that complemented the discussions among HCPs. However, these views should not be considered representative of the broader patient population.

### Thematic Analysis

#### Overview

The following quote is a descriptive metaphor for how SM is perceived by the study participants, illustrating the dynamic roles between HCPs and individuals with SM practices.

*The patient is the one at the [steering] wheel of the train and we are really just train conductors. We can point out the right switch, the right track, put the train back on track, but the patient has to drive themself.... [W]e have to teach them how to drive a train, of course*.

[Participant L, diabetes educator]

This narrative captures the central characteristics of SM: patients are the main players in managing their condition, while HCPs serve as supporters and guides in the process. The metaphor of the train reflects the long-term and unpredictable nature of T2DM management. The inductive thematic analysis resulted in the identification of the following 5 overarching themes with a summary in Table 2.

**Table 2.** Five main themes and subthemes reflecting the main study findings.

Themes	Subthemes
Opportunities to improve self-management practices	
Theme 1: Empowerment Through Education	Patient education forms the foundation for effective self-management Patients are not experts of self-management at diagnosis and require education Patients are at the center of self-management but require adequate support Peer-to-peer education is beneficial but should be supplementary to the professional advice
Theme 2: Tailoring Education to Individual Needs	Education should be tailored to the diverse diabetes population Patient willingness and engagement are critical to the success of self-management Holistic approaches should be included in education and care
Theme 3: Multidisciplinary and Cross-Sectoral Collaboration	A unified multidisciplinary team of health care professionals is necessary to support and guide patients The multidisciplinary team should be tailored to the individuals’ needs Effective communication and role clarity are facilitators for cross-sectoral and multidisciplinary collaboration Communication is key in collaboration, and data sharing could enhance this Data sharing could exacerbate the overburden of the professionals
Health ICT^a^ to improve self-management	
Theme 4: Opportunities for Digital Technology in Self-Management	Digital technology can improve self-management and improve health literacy Several features can be included in potential digital tools to improve self-management These technologies must be tailored to the individual, the context, and remain user-friendly
Limitations and threats to improving self-management practices	
Theme 5: Regional and National Systemic Limitations	There are several limitations beyond the patient and providers’ control (Primary) health care professionals are overburdened The paid-per-service model in primary care limits tailored education and care Legal and regulatory barriers limit the possibility of data sharing within the health care provider team There is a lack of digital infrastructure to accommodate digital innovations

a ICT: information and communication technology.

#### Theme 1: Empowerment Through Education

##### Individuals Require Training Programs to Drive Their Train on a Lifelong Journey of Diabetes

According to the participants, the practice of SM or “driving the train” starts with a strong foundation in patient education. Empowering individuals through education and improving health literacy were described as essential first steps in adopting effective SM, especially given the lifelong nature of the T2DM chronic disease and the frequent lack of understanding and insight the patients have about the condition. Participants explained that at diagnosis, individuals with T2DM are not automatically equipped with the knowledge or skills to manage their condition and must be trained and supported in learning how to integrate appropriate SM practices into their daily routine.

While the patient remains at the center of this process and “has to drive the train,” actively engaging in their care, HCPs must play a guiding role. Their support remains crucial in preparing patients to take on the daily responsibility of managing their condition.

*I think a problem is that, the insight into the disease is often lacking. Ultimately I think it’s [also] very important that...from the moment patients are diagnosed with diabetes, they need to be well aware of it and education by the various health care providers needs to be put in motion.*

[Participant G, pharmacist]

An interesting observation during the discussions was the resistance from the participants regarding peer-to-peer education. Some stakeholders explained that allowing people with lived experience and knowledge to support others, as a supplement to professional medical care, could be beneficial to sharing (daily) experiences. However, the majority expressed strong reservations. They argued that only trained medical professionals are adequately equipped to provide safe, accurate, and consistent education on SM, as the T2DM trajectories vary among individual patients. Concerns were raised about quality control and the risk of misinformation if peer-led education were to replace or overshadow professional guidance.

*I don’t think I would be in favor of...advice from other patients.... I am of the principle that a cobbler has to stick to their last. So I’m going to believe my doctor, my educator, my specialist but someone else? No, sorry.*

[Participant A, individual with lived experience]

*Peer to peer [education] seems indeed very useful to me. They can exchange experiences in relation to the day-to-day things. But in terms of medication use and what’s going on in the body, it really should be done by a professional.*

[Participant R, nursing diabetes educator]

### Theme 2: Tailoring Education to Individual Needs

#### Different Drivers and Train Models Require Unique Training Programs

Building on their mutual agreement that education and health literacy are fundamental for effective SM practices, stakeholders emphasized that health education and training must be tailored and personalized to the individual’s condition, capacity, and social context. They stated that tailoring educational approaches could accommodate the generational differences within the Belgian diabetes population. The population was described as highly heterogeneous, including multiple age groups with differing learning preferences and varying levels of (digital) literacy.

*[W]e are talking about a very large and very diverse population in which the education must also be adapted to their health skills and health literacy. We are now also noticing (that) within the organization that you cannot give the same education to everyone. So, the education has to be adapted to the highly educated versus the less educated, to the available health skills. And I think yes, that’s also going to have its effects on the self-management of that person.*

[Participant D, GP and researcher]

*I just wanted to add that education is also tailoring. And also choosing the right form of work.... [I]t’s just tailor-made*.

[Participant O, dietitian and researcher]

Furthermore, the patient’s willingness, motivation, and readiness to engage were also viewed as critical to the success of any SM strategies, and therefore, health information should be delivered in a way that aligns with their goals, needs, and values. In other words, education must allow individuals to “individualize their training, according to their train model.”

*But above all, it is also yes, that openness on the part of the patient, being open to it,... to all the education that comes with it*.

[Participant K, nursing diabetes educator]

Several participants also claimed that effective education must be extended beyond clinical and medical and paramedical knowledge. They advocated for a holistic, person-centered approach to care and education, where the main goal should be quality of life. They agreed that educational approaches should not only address medical aspects but also include the emotional, social, and psychological elements of this chronic illness.

*But we also have to look more holistically, more at the psychological and emotional aspects. And also the social roles, and take them into account in the care process, whereby the patient’s goals, wishes, and strengths are actively included in the care process.*

[Participant J, researcher]

### Theme 3: Multidisciplinary and Cross-Sectoral Collaboration

#### The Drivers Require a Team of Train Conductors During the Journey

In the opening quote, a diabetes educator compared the role of providers in the SM process with that of “train conductors,” guiding and supporting patients along this journey. Participants strongly agreed that effective diabetes care and support requires a unified, multidisciplinary team involving a diverse group of medical professionals, including GPs, endocrinologists, diabetes educators, dietitians, nurses, and psychologists. However, they explained that not every professional needs to be actively involved in every case; rather, the composition of the team should be tailored to the individual needs and context of each patient.

Two key facilitators of coordinated collaboration across disciplines and sectors emerged from the discussion: role clarity and effective communication. First, stakeholders noted that the current engagement of providers is uneven, largely due to unclear role definitions within the multidisciplinary team. For example, GPs are typically referred to as the “gatekeepers” of care, while diabetes educators are expected to serve as central actors in supporting SM. In practice, however, the fragmented care landscape usually prevents patients from accessing educators, and GPs, faced with time constraints and huge workloads, may not be able to fully assume this guiding role. As a result, some endocrinologists are stepping into positions typically reserved for primary care, highlighting the need for a more integrated and coordinated approach to interprofessional collaboration across the primary, secondary, and tertiary care settings.

*It’s not the patient by themself, but it’s actually multidisciplinary. You have to work together with the endocrinologist, GPs, the educator, dietitians...*

[Participant C, nursing diabetes educator]

*Everyone [HCPs] can give a piece of education, but there has to be a very clear agreement about it. Who takes on which role? I think that will only lead to greater job satisfaction and also, I think, happier patients if they see that there is also good cooperation.*

[Participant F, GP and researcher]

Second, participants agreed that clear and consistent communication is critical to the success of multidisciplinary and cross-sectoral collaborations. Unified and nonconflicting messaging is essential to avoid confusion and ensure that patients receive coherent advice and support across the care settings. Participants further noted that improving data transparency and accessibility could play a pivotal role in improving collaboration and coordination, which is further discussed in the following subtheme.

*[C]ommunication is so very important and that it actually all comes down to communication.*

[Participant I, GP]

*If all those health care providers then indeed also put forward the same story, then the patient eventually starts to feel that too, and indeed they do feel empowered by the whole multidisciplinary team standing around him.*

[Participant G, pharmacist]

#### Subtheme: Enhancing Collaboration Through Data Sharing

##### Conductors Must Be Well Informed to Ensure Safe and Efficient Operation of the Train

Experts explained that limited data sharing among HCPs is a significant barrier to delivering continuous, coordinated care, and ultimately compromises efforts to support SM for individuals with T2DM. One clear example raised was the lack of access to electronic medical records (EMRs) across the disciplines: diabetes educators or dietitians often cannot view physicians’ EMR, while physicians also lack access to relevant paramedical data. This digital data siloing and fragmentation undermines communication and reduces the ability to provide personalized support. Participants advocated for the development of a centralized or transparent data-sharing platform that would allow providers access to essential patient medical records. Importantly, the participants also remarked that public attitudes toward sharing health data are generally positive, especially if this is used to improve care in general.

*I think there are gaps on the sharing of data: the medical data and paramedical data. What did the home nurse or the dietitian in the first or second line say or do, what might that GP have done? We don’t have access to the GP’s data or file. We miss a lot of information there.*

[Participant P, endocrinologist]

*[A] centralized or a common system where everyone can have their input. I think self-management could be much better if indeed such a system existed.*

[Participant B, GP and researcher]

### Theme 4: Opportunities for Digital Technology in SM

#### Innovative Tools Can Improve the Train Journey

Stakeholders broadly agreed that digital technology holds great potential to enhance SM and improve care experience for patients. They expressed strong support for the integration of digital tools and supporting digital infrastructure as a way to promote SM behaviors, improve health literacy, and the overall management of T2DM.

According to participants, digital platforms and health ICT could offer new opportunities to connect providers with other providers and patients, facilitate the ongoing support and address gaps in diabetes education. These tools were described as valuable for tailoring education to individual needs, offering efficient health data visualization, and improving the continuity and coordination of care. In addition, participants explained that with such opportunities, patients could be empowered to interpret their own health data, which could reduce the workload for GPs and other providers, while also guiding patients to find other providers within the multidisciplinary team for specific concerns. The common features they referred to included digital tools with real-time monitoring, visual data display, automated and semiautomated applications, dashboards, and reminder functions to support day-to-day management of diabetes.

*So personally for self-management, I think it would be ideal should there be an app that would help me towards the future, to have certain things replenished or adjusted in time: for example you’d best consult an educator now.*

[Participant A, individual with lived experience]

However, participants emphasized that these tools must also be tailored to the specific needs of the patient population, taking the varying levels of patient and professional digital literacy into account. Accessibility and user-friendliness were also named as potential facilitators for effective integration of digital innovation into diabetes care. Finally, while supportive of digital innovation, the stakeholders agreed that such technologies should complement and not replace human interaction, professional expertise, and medical reports formally written by physicians.

*I think technology can always be a very big supportive factor in that [SM], as long as it’s very tailored and very user-friendly. I think that’s kind of where the challenge lies now, to make sure that everybody is along in that digitalization.*

[Participant M, medical ICT expert and developer]

### Theme 5: Regional and National Systemic Limitations

#### Train Tracks, Stations, and Other External Conditions Affect the Journey, Beyond the Driver’s Control

While the first 4 themes reflected on opportunities to strengthen SM practices in Flanders, Belgium, participants also identified several systemic limitations that indirectly affect the SM landscape. The first concern was how to scale up tailored education without overburdening the professionals or exacerbating the already fragmented care landscape. GPs, who were referred to as “gatekeepers,” are overwhelmed by high workloads, reducing their ability to proactively support patients with T2DM (and other chronic diseases). Referring patients to diabetes educators could ease this pressure, but participants warned that it would only shift the burden, as there are too few educators to meet this high demand, especially when repeated support and tailored education are required. Additionally, stakeholders explained that the current limit on the number of reimbursed diabetes education sessions in the primary care system also creates a structural barrier to providing patient-centered, individualized education. Because educators are compensated per session and only up to a fixed number of sessions per patient, there is limited flexibility to offer multiple, repeated, or extended tailored sessions based on the patients’ needs.

*Actually...if you start education in diabetes, the educator should...organize that. If they thinks they [the patient] needs to see a pharmacist for therapy compliance and so on, they should gather them [HCPs] around them [the patient] and possibly organize group education around it. But now...we lack an organizational structure, in first line. In the second and third line, it is already much easier, those people are in our corridor, we can meet with each other, but in the first line it is not like that.*

[Participant Q, endocrinologist]

Legal and regulatory barriers also emerged as a threat. Data protection regulations currently restrict access to medical data for certain members of the multidisciplinary team, such as dietitians, psychologists, and physical therapists. Although these professionals play vital roles in the multidisciplinary team, their limited access to relevant patient data undermines integrated care delivery.

*One of the reasons why dietitians for example are not allowed to see labs [results] is because it is not legally allowed. That’s not just digital barrier, but a legal one. Some health care professions have access—others don’t.*

[Moderator 2, endocrinologist]

Finally, participants pointed to the lack of digital infrastructure across health care institutions in Flanders that could permit interoperable or exchangeable systems as a major obstacle to digital innovation. Additionally, digital tools that would offer real-time monitoring of patient data would require human control or oversight. Given the overburdened HCPs, stakeholders questioned who would be responsible for monitoring all that incoming data.

*Second thing I wanted to come back to briefly is on digital evolution. A lot is happening at the moment.... And we are now talking about one platform or one server that holds all that data, right? I don’t know whether that’s really the way to go and whether it’s going to be feasible to do it that way. It’s going to be very tricky to bring all the data together in one place from all diabetes patients—pretty tricky, certainly in terms of security. What I think is a better path is actually the exchangeability of the data, the interoperability to start with.*

[Participant F, GP and researcher]

## Discussion

### Principal Findings

This exploratory qualitative study identified five interconnected themes describing stakeholders’ perspectives on improving diabetes SM and the potential role of digital health technologies in supporting SM practices within the Flemish health care system: (1) Empowerment Through Education, (2) Tailoring Education to Individual Needs, (3) Multidisciplinary and Cross-Sectoral Collaboration, (4) Opportunities for Digital Technology in Self-Management, and (5) Regional and National Systemic Limitations. Collectively, these findings illustrate that stakeholders perceived diabetes SM as extending beyond patients’ individual behaviors and involving interactions between patients, HCPs, and the broader health care system.

At the patient level, stakeholders consistently described effective SM as depending on patients’ motivation, readiness, and engagement in their care. However, they also emphasized that these capabilities are influenced by the availability and quality of tailored education, continuous support provided by HCPs, and approaches that acknowledge patients’ individual preferences, skills, and psychosocial needs. Given that only 1 individual living with T2DM participated in this study, these findings should be interpreted as stakeholders’ perceptions of the factors that support patient SM rather than as a direct representation of patients’ experiences.

At the HCP level, participants highlighted the central role of HCPs in facilitating SM through person-centered education, multidisciplinary collaboration, and coordinated care across health care settings. Simultaneously, stakeholders described substantial practical barriers, including increasing workload, limited consultation time, fragmented communication between care sectors, and organizational constraints that reduce opportunities to provide personalized support. Participants also recognized that different HCPs contribute distinct expertise to diabetes management, and that improving SM does not necessarily require every professional or digital solution to fulfill the same function. Rather, complementary roles within a coordinated multidisciplinary team were considered essential.

The HCPs and other stakeholders described organizational, legislative, and technological factors that influence the delivery of diabetes care at the regional and national health care system level. These included reimbursement structures, legal restrictions on data sharing, workforce capacity, and digital infrastructures. Although many stakeholders considered improved interoperability and data exchange desirable to support communication and continuity of care, the discussions also recognized that different solutions may appropriately serve different purposes within different health care settings. Rather than advocating for 1 fully integrated system, participants emphasized the importance of digital technologies that are interoperable, user-friendly, fit their intended clinical context, and complement existing care processes.

While previous (qualitative) studies have explored diabetes SM and the role of digital health technologies in other health care settings, this study contributes context-specific insights into how stakeholders within the Flemish health care system perceive opportunities and barriers to improving diabetes SM and the potential role of digital tools. The findings demonstrate that although many challenges identified internationally, such as the need for personalized education, multidisciplinary collaboration, and user-friendly digital solutions, are also recognized in Flanders, stakeholders consistently highlighted that their successful implementation depends on addressing context-specific barriers, including fragmented digital infrastructure, limited interoperability between health care sectors, and organizational constraints within the Belgian health care system.

### Comparison With Literature

Diabetes SM has been defined as a set of activities, including medication adherence and lifestyle modifications, undertaken by patients with guidance from HCPs. This term is used interchangeably with self-care, as they are similar yet with distinct components of diabetes care, and therefore during the discussion, findings were compared with existing literature on SM and self-care practices for diabetes and chronic diseases [41]. Consistent with previous research, stakeholders emphasized that effective SM requires sufficient diabetes-related knowledge, skills, and empowerment [42]. The American Diabetes Association underscored the importance of education in SM, noting that it enhances confidence, improves health outcomes, and increases the capacity of individuals to make informed daily decisions. Similarly, educational interventions have demonstrated significant improvements in SM behaviors and glycemic outcomes [42,43]. However, our findings further demonstrate that, within the Flemish health care context, education alone is perceived as insufficient unless accompanied by coordinated multidisciplinary support and health care structures that enable personalized care. Sustained professional and social support, motivation, and a positive attitude toward SM have been described as key predictors for long-term adherence to SM [44-46]. The study findings reflect these results, characterizing SM as a patient-centered yet professionally supported process.

Social support, particularly from family and peers, has consistently been associated with improved SM [47-49]. While some stakeholders expressed reservations, others were opposed to peer-to-peer education. They, however, acknowledged its potential value as a complement to professional guidance. Other studies, however, support this and several peer-led programs have demonstrated benefits in reduced diabetes distress and patient empowerment, even when the intervention had moderate effects on HbA_1c_ values [47-49]. More than 60% of the study participants were older than 40 years, and this demographic composition may have shaped the generally cautious views on peer support; perspectives might differ in a younger cohort.

Tailoring education and care for individual needs was another recurrent theme. Personalized approaches that incorporate patients’ goals, skills, and personal context have been shown to enhance SM and clinical outcomes [50-52]. Digital interventions offering individualized feedback and goals, targeted approaches, and adaptive features have similarly led to improved SM practices and HbA_1c_ levels [53-55]. This supports stakeholders’ views that education, multidisciplinary teams, and digital tools should be tailored and person-centered.

Consistent with study participants’ analogy of HCPs as “train conductors” rather than drivers, previous research describes HCPs as facilitators of SM who support patients in developing knowledge, skills, and confidence needed to participate actively in their care [56]. Multidisciplinary and interprofessional collaboration across primary, secondary, and tertiary care has also been associated with greater patient satisfaction and improved SM behaviors [43,57,58]. However, participants in this study identified heavy workloads, limited consultation time, and organizational constraints as barriers to providing personalized education. Maintaining effective communication channels, shared care plans [59,60], clear documentation, regular multidisciplinary meetings, and joint goal setting may help address these barriers and facilitate more coordinated SM support [57,59,61-63].

Participants also viewed digital technologies as potential enablers of both patient empowerment and collaboration among HCPs. Previous research suggests that digital systems incorporating real-time monitoring, graphical data visualization, personalized feedback, and integrated communication functions may support patient engagement and clinical outcomes [22,23,60]. However, our findings suggest that the perceived value of such technologies depends partly on their fit with existing clinical workflows, the extent to which relevant information can be accessed across health care settings, and organizational readiness within the Belgian health care system. Participants highlighted fragmented digital infrastructure and limited interoperability between EMRs across health care sectors as important contextual barriers to effective information exchange [64-66]. Similarly, a 2024 MedTech Europe report identified the absence of harmonized legislation in Europe and limited access to EMRs among paramedical professionals as barriers to coordinated, multidisciplinary diabetes care [67]. Although initiatives such as the Belgian Integrated Health Records [68] aim to improve health information exchange, participants’ accounts indicate that the challenges in achieving effective interoperability and across-sectoral information sharing remain relevant to the implementation of digital health solutions.

### Strengths and Limitations

A major strength of this study lies in the choice of a rigorous analytic approach. The semistructured discussion guide was informed by the CFIR [25] and evidence on diabetes SM and digital health interventions, ensuring that the discussions were theoretically grounded while remaining sufficiently flexible to explore emerging topics relevant to the Flemish health care context. Data were analyzed using Braun and Clarke’s 6-phase thematic analysis [38,39], following an iterative and transparent analytical process. Independent coding by 2 researchers (BA and PVB) was applied to interpret the findings from the focus group discussions. Thematic data coding was complemented by reflexive discussions to compare interpretations, resolve discrepancies, and refine the developing themes. The second coder (PVB) was not involved in the study or guide design, participant recruitment, or data collection, providing an independent analytical perspective that was unshaped by the earlier stages of the study and thereby strengthening the credibility of the findings. Emerging themes were subsequently discussed within the multidisciplinary research team to ensure that interpretations remained grounded in the data.

Several additional strategies were used to enhance the trustworthiness of the study. Maximum variation purposive sampling facilitated the inclusion of participants with diverse professional roles, health care settings, and levels of experience, while the heterogeneous composition of the focus group promoted interaction and dialogue between stakeholders from different sectors of diabetes care. During the focus group discussions, 2 members (BA and CJ) of the research team observed participant interactions and documented potential nonverbal cues to support interpretation of the transcripts. Furthermore, credibility, dependability, transferability, and confirmability were strengthened through investigator triangulation, documentation of the analytical process, reflexive team discussions, and transparent reporting in accordance with the COREQ checklist [29]. Collectively, these methodological strategies enhance confidence in the credibility and transferability of the study findings to similar health care settings.

Several limitations should be considered when interpreting the findings of this study. The principal limitation of this study is the limited representation of people living with type 2 diabetes. Although this study was intentionally designed to include multiple stakeholder groups, only 1 individual with lived experience ultimately participated in the focus group discussions. Consequently, the findings primarily reflect the perspectives of HCPs, researchers, and digital health stakeholders regarding how diabetes SM could be improved, rather than providing a comprehensive understanding of patients’ own experiences, priorities, and expectations. Given that SM (and self-care) is inherently centered on the individual’s lived experience, motivation, and daily decision-making [41], important patient perspectives may therefore be underrepresented in this study. Furthermore, the participant with lived experience contributed within a multidisciplinary focus group that included HCPs, one of whom is also involved in their clinical care. Although the moderator actively encouraged balanced participation throughout the discussion, this group composition may have influenced the participant’s willingness to openly discuss personal experiences, perspectives, or views that differed from those of the professionals. Consequently, the patient perspective presented in this study should be interpreted cautiously and viewed as complementary to, rather than representative of, the broader patient experience. Additionally, only 2 discussions were conducted. Although substantial thematic overlap was observed across both discussions and the data provided sufficient depth to support the identified themes, additional focus groups or individual interviews may have generated further nuances or perspectives relevant to diabetes SM and digital health implementation.

A further limitation relates to the recruitment strategy. HCPs were initially recruited through the research team’s professional networks and expanded using snowball sampling, while people living with T2DM were primarily recruited through participating HCPs. Although these approaches were appropriate for identifying information-rich participants with relevant expertise, they may have introduced selection bias by favoring individuals who were professionally connected to the research team or already engaged in diabetes care. Consequently, some perspectives, particularly those of less engaged HCPs or people with different experiences of diabetes care, may not have been represented.

Furthermore, no formal participant validation (member checking) was conducted. Participants were informed of the final study findings after completion of the analysis. They were not invited to review the transcripts or comment on the preliminary themes during the analytical process. Consequently, participants did not have the opportunity to confirm whether the researchers’ interpretations reflected their intended meanings. To enhance the trustworthiness of the findings, we instead used investigator triangulation through independent coding by 2 researchers (BA and CJ), iterative theme refinement, reflexive discussions within the multidisciplinary research team, and transparent reporting according to the COREQ guideline.

As the focus group discussions were conducted in Dutch and analyzed using English translations, subtle linguistic and cultural nuances may have been lost during translation [69]. To minimize this risk, all translated transcripts were independently reviewed against the original Dutch transcripts by native Dutch- and English-speaking members of the research team, no discrepancies were identified. In addition, although the semistructured discussion guide was informed by the CFIR framework, it was not formally piloted prior to data collection. Pilot testing may have identified opportunities to further refine the wording, sequencing, or clarity of the discussion questions [70,71].

The online format of the focus groups, while practical for the participants and organizers, may have affected the depth of engagement, interaction, and group dynamics, which is another limitation to be considered. Nonverbal cues were reduced and some technical difficulties such as background noise, frozen screens, and unstable internet connections may have disrupted the flow of discussions. This format may also have excluded individuals with limited digital access, literacy, or skills, potentially narrowing the diversity of perspectives, especially regarding the use of digital tools and ICT.

Findings from this study should therefore be interpreted as reflecting stakeholders’ perspectives on improving diabetes SM within the Flemish health care context, with particular emphasis on the views of HCPs, rather than as representative of the experiences of people living with T2DM. Future qualitative research should purposively recruit a larger and more diverse sample of people living with type 2 diabetes by combining recruitment through patient organizations, primary care networks, professional societies, diabetes associations, and other community-based recruitment pathways. Such strategies may improve the diversity and representativeness of patient perspectives while reducing potential recruitment bias. In addition, patient-only focus group discussions or individual interviews should be considered to facilitate open discussions of lived experiences, SM practices, and support needs. Comparing these findings with those of HCPs and other relevant stakeholders would provide a more comprehensive understanding of the opportunities, barriers, and contextual factors influencing the improvement of diabetes SM, as well as the potential role of digital tools in supporting these practices.

### Reflexivity

The multidisciplinary research team comprised researchers with expertise in general practice, biomedical sciences, endocrinology, diabetes care, chronic disease management, EMRs, and clinical audit and feedback. These complementary clinical and research backgrounds enriched the interpretation of the study data collection and analysis. At the same time, the predominant clinical backgrounds of the research team may have influenced the interpretation of themes, particularly those relating to health care delivery, multidisciplinary collaboration, and the implementation of digital health technologies.

The focus group discussions were moderated by GG, an experienced GP and researcher with more than 30 years of clinical and academic experience. He also assisted in the recruitment of many of the participating HCPs through existing professional networks. Although no prior researcher-participant relationship existed for most participants beyond these professional contacts, this dual role may have influenced participants’ willingness to express opinions that differed from prevailing clinical perspectives or from those of the research team. To minimize this potential influence, participants were informed that there were no right or wrong answers, that all viewpoints were valued, and that discussions would remain confidential. During the focus groups, the moderator actively encouraged balanced participation by inviting quieter participants to contribute and ensuring that no individual or professional group dominated the discussion. Particular attention was given to encouraging the participant living with T2DM to share personal experiences throughout the discussion.

The study was conducted within the broader DIPAR project, which aims to develop and evaluate a digital intervention to support diabetes care. Consequently, the research team entered the study with an existing interest in understanding how digital interventions could support diabetes SM. This prior engagement may have increased the researcher’s sensitivity to identifying opportunities for digital tools during data collection and analysis. However, the discussion guide was intentionally designed to explore stakeholders’ perspectives on diabetes SM more broadly, including existing practices, barriers, and facilitators, rather than assuming that digital technologies represented the preferred or only solution. Throughout the analysis, themes were developed inductively from participants’ accounts rather than being mapped onto predetermined categories such as the CFIR framework for intervention implementation or others related to digital health.

The primary coder (BA), a biomedical scientist and PhD researcher investigating the use of digital tools to support the management and process of care in type 2 diabetes, and the second coder (PVB), a GP and PhD researcher focusing on shared decision-making in medical education, independently coded the transcripts before comparing interpretations through reflexive discussions. PVB has prior experience with qualitative synthesis but had not been involved in the study design, participant recruitment, and focus group facilitation, thereby providing an independent analytical perspective during theme development.

Emerging interpretations were subsequently discussed within the multidisciplinary research team, where alternative explanations were actively considered and themes were refined through consensus to ensure that the final interpretations remained grounded in the participants’ accounts rather than the researchers’ preconceptions. The team aimed to approach the study with openness and curiosity toward exploring stakeholders’ perspectives on how SM practices could be improved in Flanders and how digital tools might support these practices. All researchers are based in Leuven, Belgium, and have experience in public health and clinical care. Consequently, the topic of improving SM was largely approached from a health system perspective, and the interpretation of findings may therefore reflect experiences rooted in this context.

### Implications for Further Research

This study aimed to explore stakeholders’ perspectives and generate insights specific to this context rather than formulating specific guidelines or policy recommendations. Nonetheless, the findings highlighted the need for future investigation. This study contributed to a further understanding of how HCPs and patients in Flanders perceive the improvement of diabetes SM processes and the 3 interconnected levels that influence this process and the integration of digital technologies to support SM.

First, future research should aim to include a broader range of HCPs such as psychologists, physical therapists, social workers, informal caregivers, and other providers that play key roles in diabetes and chronic disease management but were not represented in our study. Second, it is essential to include more individuals with T2DM in focus groups within a similar context. Participants should reflect diverse socioeconomic, cultural, and educational backgrounds to ensure a more comprehensive representation of the patients’ perspectives.

In addition, the influence of organizational and policy-level factors on implementation remains insufficiently explored in this study. The relationship between the interconnected operating levels that influence SM also remains insufficiently explored in this paper. Future studies could explore how the identified themes may be operationalized and implemented using established implementation frameworks and subsequently evaluated through mixed methods and quantitative research to assess their effectiveness in improving SM in real-world health care settings.

### Conclusions

This qualitative study explored stakeholders’ perspectives on improving SM for people with type 2 diabetes within the Flemish health care context and assessed the potential role of digital health technologies in supporting these practices. Five interconnected themes were identified: Empowerment Through Education, Tailoring Education to Individual Needs, Multidisciplinary and Cross-Sectoral Collaboration, Opportunities for Digital Technology in Self-Management, and Regional and National Systemic Limitations. Together, these findings highlight that effective SM may extend beyond individual patient behaviors and could be shaped by interactions between patients, HCPs, and the broader health care system. While digital technologies were perceived as valuable enablers of SM, participants emphasized that their successful implementation depends on addressing organizational and contextual challenges. Further qualitative research should include larger and more diverse patient populations to further explore lived experiences and inform the development of person-centered, contextually appropriate digital SM interventions.

## Supplementary material

10.2196/88446 Multimedia Appendix 1 Information and consent form.

10.2196/88446 Multimedia Appendix 2 Interview guide and demographic questionnaire.

10.2196/88446 Checklist 1 COREQ checklist.

## Abbreviations

CFIR: Consolidated Framework for Implementation Research
COREQ: Consolidated Criteria for Reporting Qualitative Research
DIPAR: Digital Diabetes Patient Reminder
DIPAR: Diabetes Patient Reminder
EMR: electronic medical record
GP: general practitioner
HbA_1c_: hemoglobin A_1c_
HCP: health care professional
ICT: information and communication technology
JACARDI: Joint Action on Cardiovascular Diseases and Diabetes
SM: self-management
T2DM: type 2 diabetes mellitus
