# Supplementary material for: Perspectives on Self-Management and Digital Tools for Individuals With Type 2 Diabetes in Flanders: Qualitative Focus Group Study
Source: JMIR Diabetes. 2026 Oct 27;11:e88446. doi: 10.2196/88446 (PMC13712406; doi:10.2196/88446)
Supplement: Supplementary file 1 [file diabetes-v11-e88446-s001.docx]

Multimedia Appendix 1

Situational analysis: Current Care Environment for patients with type 2 diabetes mellitus in Flanders

Information and Consent Form

Version date: 16^th^ May 2024

Version 3

**Title of the research**: Situational analysis of the Current Care Environment for patients with type 2 diabetes mellitus in Flanders

**Sponsor of the study:** The Digital Diabetes Patient Reminder Tool pilot project, part of the Joint Action for Cardiovascular Diseases and Diabetes and funded by the European Union

**Medical Ethics Committee:** Research Ethical Committee UZ/KU Leuven

**Name + contact details of supervisor and researcher(s):**

Barbara Annancy ([barbara.nii-annancy@kuleuven.be](mailto:barbara.nii-annancy@kuleuven.be))^1^

Geert Goderis ([geert.goderis@kuleuven.be](mailto:geert.goderis@kuleuven.be))^1^

Roman Vangoitsenhoven ([roman.vangoitsenhoven@kuleuven.be](mailto:roman.vangoitsenhoven@kuleuven.be))^2^

Steve Van den Bulck ([steve.vandenbulck@kuleuven.be](mailto:steve.vandenbulck@kuleuven.be))^1^

^1^Academic Center for General Practice 
Department of Public Health and Primary Care, KU Leuven 
Kapucijnenvoer 7 blok h, 3000 Leuven, Belgium

^2^Clinical and Experimental Endocrinology, UZ Leuven 
UZ Herestraat 49 - bus 7003, 3000 Leuven, Belgium

## I. **Information vital to your decision to take part**

**Introduction**

You are being invited to take part of a qualitative study in the form of a stakeholder discussion. This means that we would like to make use of your knowledge and insights into the current setting in Flanders of self-management for people with type 2 Diabetes Mellitus (T2DM) and how the development of an innovative digital tool could support and empower people with T2DM, in accordance with the conditions of good medical practice and independently of your possible participation in this study.
We are simply asking you whether we can collect data from your statements and opinions that will be uttered during the stakeholder discussion and process them statistically for research purposes.
No additional diagnostic or monitoring procedure will be proposed.

Before you agree to take part in this study, we invite you to take note of its implications in terms of organisation, possible risks and benefits, to allow you to make a decision with full awareness of the implications. This is known as giving “informed consent”.

Please read these few pages of information carefully and ask any questions you want to the investigator or his/her representative.

There are 3 parts to this document: the information essential to your decision, your written consent and supplementary information (appendices) detailing certain aspects of the basic information.

**If you take part in this study, you should be aware that:**

- No form of treatment will be offered to you by the investigator during this study.
- This clinical study is being conducted after having been reviewed by the Ethical Committee of the UZ/KU Leuven.
- Your participation is voluntary and must remain free from any coercion. It requires the signature of a document expressing your consent. Even after having signed this document, you can stop taking part by informing the investigator.
- The data collected on this occasion are confidential and your anonymity is guaranteed during publication of the results.
- You may contact the investigator or a member of his/her team at any time should you need any additional information.

Further information about your “Rights as a participant in a clinical study” can be found in III. Supplementary information on page 5.

**Objectives and course of the study**

This clinical study has been organised to gain insight and understanding into the current state of self-management for people with type 2 diabetes mellitus (T2DM) in Belgium and how a digital, targeted at both the healthcare providers and people with T2DM, can help empower, improve and facilitate the self-management processes.

We are inviting you to take part in this clinical study because of your current job function and expertise regarding the objectives within the context of this study. This clinical study is to include about 20 participants, all from the Flanders region in Belgium.

To be able to take part in this study you must be active as:

- Diabetologist/Endocrinologist
- General Practitioner
- Experts with lived experiences
- Pharmacist
- Diabetes Educators
- Medical ICT expert and technology specialists
- Dietitians
- Nurses (in the primary and secondary healthcare setting)
- Organizers of patient organizations

Inclusion criteria

- Experts by experience aged 18 years or above and being treated for T2DM.
- Experts by experience considered clinically well to participate, determined by core research team.
- Family members or informal caregivers with experience supporting the care for individuals with T2DM aged 18 years or above.
- Working with or treating individuals with T2DM
- Understanding and speaking Dutch or English

Your participation in the stakeholder discussion will last the length of a single focus group discussion (between 90 – 120 minutes), which will include about 10 participants per session. During the discussion session the moderator will raise open-ended questions regarding the current state of self-management for people with T2DM, the use of ICT to support and facilitate the self-management and finally questions regarding the facilitators and barriers of an innovative digital tool to be implemented in Flanders and utilized by both healthcare professionals and patients of T2DM. Also prior to the discussion a brief questionnaire will be filled in to gather demographic data of all participants including factors such as age, gender, job function and work location in Flanders.

**Description of risks and benefits**

No risk, in terms of health, can be linked to your participation in this study.

Similarly, you should not expect any personal benefits as a result of taking part in the study. Know only that your participation will allow us to better understand how the self-management environment in Flanders can be improved and what the facilitators and barriers are for the development of the afore mentioned innovative digital tool, thus to improve the self-management processes in the future.

**Withdrawal of consent**

Your participation is voluntary and you are entitled to withdraw your consent to take part in the study for any reason, without having to justify your decision.

If you withdraw your consent to take part in the study, to guarantee the validity of the research, the data encoded up to the point at which you withdraw will be retained. No new data may be sent to the sponsor. The sponsor/party responsible for the study may also decide to stop the study because the data collected provide a faster response than originally expected.

**If you take part in this study, we ask you:**

- To cooperate fully in the smooth running of this study.
- Not to conceal any kind of information of importance to our study.

### *Contact*

- If you need further information, but also if you have problems or concerns, you can contact the investigator (Goderis, Geert) or a member of his research team (Nii-Annancy, Barbara)

Prof Geert Goderis – [geert.goderis@kuleuven.be](mailto:geert.goderis@kuleuven.be), +3216377280

Barbara Nii-Annancy – [barbara.nii-annancy@kuleuven.be](mailto:barbara.nii-annancy@kuleuven.be), +3216330078

This study has been reviewed and approved by the Ethics Committee Research at the UZ / KU Leuven (approval number). If you have any questions relating to your rights as a participant in a clinical study, you can contact the Ethical Committee Research UZ/KU Leuven ([ec@uzleuven.be](mailto:ec@uzleuven.be); 016 34 86 00 (workday between 10h and 11h))

Finally, if you have a complaint about the processing of your data, you can contact the Belgian supervisory body that monitors compliance with the fundamental principles of personal data protection, the Belgian supervisory authority is called:

Gegevensbeschermingsautoriteit (GBA)

Drukpersstraat 35,

1000 Brussel

Tel. +32 2 274 48 00

e-mail: contact(at)apd-gba.be

Website: [www.gegevensbeschermingsautoriteit.be](http://www.gegevensbeschermingsautoriteit.be/)

**Title of the research**: Situational analysis of the Current Care Environment for patients with type 2 diabetes mellitus in Flanders

## II. **Informed Consent**

**Participant**

- I declare that I have been informed of the nature of the study, its purpose, its duration, the possible side effects and what is expected of me. I have taken note of the information document and the appendices to this document.
- I have had sufficient time to think about it and discuss it with a person of my choice.
- I have had the opportunity to ask any questions that came to mind and have obtained a favourable response to my questions.
- I understand that data about me will be collected throughout my participation in this study and that the investigator and the sponsor of the study will guarantee the confidentiality of these data in accordance with applicable European and Belgian legislation.
- I agree to my personal data being processed as described in the section dealing with confidentiality guarantees (page 5-6).
- I want/don’t want to be informed by the overall research results from this study.
- I have received a copy of the information to the participant and the informed consent form.

Surname: ……………………………………………………..

First name: ……………………………………………….…..

Date: ……………………………………………………………

Signature of the participant: ……………………………..

**Investigator**

- I, the undersigned prof Geert Goderis / Barbara Nii-Annancy investigator, confirm that I have verbally provided the necessary information about the study and have given the participant a copy of the information document.
- I confirm that no pressure was applied to persuade the participant to agree to take part in the study and that I am willing to answer any additional questions if required.
- I confirm that I operate in accordance with the ethical principles set out in the latest version of the “Helsinki Declaration”, the “Good Clinical Practices” and the Belgian Law of 7 May 2004 related to experiments on humans.

Name, date and signature of the investigator:

………………………………………………………

**Title of the research**: Situational analysis of the Current Care Environment for patients with type 2 diabetes mellitus in Flanders

## III. **Supplementary Information**

**1: Supplementary information on the organisation of the study**

Participants will only be invited to one of the stakeholder discussion. This will take place through the Teams communication online platform, and will last anywhere between 90 – 120 minutes. A maximum of 10 participants will be invited per discussion to ensure that all participants can partake in the discussion.

**2: Supplementary information on the protection and rights of the participant in a clinical study**

### *Ethics Committee*

This study has been reviewed by an independent Ethics Committee, namely the Ethics Committee of Ethical Committee Research UZ/KU Leuven, which has issued a favourable opinion. It is the task of the Ethics Committees to protect people who take part in a clinical trial. They make sure that your rights as a patient and as a participant in a clinical study are respected, that based on current knowledge, the study is scientifically relevant and ethical.
You should not under any circumstances take the favourable opinion of the Ethics Committee as an incentive to take part in this study.

### *Voluntary participation*

Before signing, do not hesitate to ask any questions you feel are appropriate. Take the time to discuss matters with a trusted person if you so wish.

Your participation in the study is voluntary and must remain free of any coercion: this means that you have the right not to take part in the study or to withdraw without giving a reason, even if you previously agreed to take part. Your decision will not affect your relationship with the investigator or the quality of your future care management.

If you agree to take part in this study, you will sign the informed consent form. The investigator will also sign this form to confirm that he/she has provided you with the necessary information about the study. You will receive a copy of the form.

***Costs associated with your participation***

You will not receive any compensation for your participation in this study. Furthermore, the study will not involve any additional costs for you.

***Guarantee of confidentiality***

Your participation in the study means that you agree to the investigator collecting data about you and to the study sponsor using these data for research purposes and in connection with scientific and medical publications. All video and audio recordings will be deleted immediately after complete transcription as in accordance with EU general data protection regulation.

Your data will be processed in accordance with the European General Data Protection Regulation (GDPR) and with the Belgian legislation on the protection of natural persons with regard to the processing of personal data. KU Leuven shall act as data controller for your data.

The name and contact of the assigned data protection officer for research at the KU Leuven is:

Name: Mr. Toon Boon

E-mail: [toon.boon@kuleuven.be](mailto:toon.boon@kuleuven.be)

Adres: Krakenstraat 3 - box 5516 3000 Leuven

Telephone: +32 16 32 40 74

You are entitled to ask the investigator what data are being collected about you and what is their use in connection with the study. This data concerns your current clinical situation but also some of your background, the results of examinations carried out within the context of care of your health in accordance with current standards. You have the right to inspect these data and correct them if they are incorrect^[1]^.

The investigator has a duty of confidentiality vis-à-vis the data collected.

The investigator and his/her team will therefore be the only ones to be able to establish a link between the data transmitted throughout the study. The personal data transmitted will not contain any combination of elements that might despite everything allow you to be identified^[2]^.

Any data transmitted will not allow you to be identified. The investigator and his team are responsible for collecting the data gathered during this study, processing them and protecting them in accordance with the requirements of the Belgian law on the protection of privacy.

If you withdraw your consent to take part in the study, to guarantee the validity of the research, the data encoded up to the point at which you withdraw will be retained. No new data may be sent to the sponsor. If you have any questions relating to how your data are being processed, you may contact the investigator.

If you subsequently have any special concerns or wish to file a complaint, please contact the KU Leuven privacy team at [privacy@kuleuven.be](mailto:privacy@kuleuven.be)

Finally, if you have a complaint about the processing of your data, you can contact the Belgian supervisory body that monitors compliance with the fundamental principles of personal data protection:

The Belgian supervisory authority is called: Gegevens Beschermingsautoriteit (GBA)

Drukpersstraat 35,

1000 Brussel

Tel. +32 2 274 48 00

e-mail: contact(at)apd-gba.be

Website: [www.gegevensbeschermingsautoriteit.be](http://www.gegevensbeschermingsautoriteit.be/)

1. These rights are guaranteed by the European Data Protection Regulation (GDPR), by the Belgian legislation on the protection of natural persons with regard to the processing of personal data and by the Law of 22 August 2002 on patient rights.
2. The database containing the results of the study will therefore not contain any combination of elements such as your initials, your gender and your full date of birth (dd/mm/yyyy).
