# Supplementary material for: Perspectives on Self-Management and Digital Tools for Individuals With Type 2 Diabetes in Flanders: Qualitative Focus Group Study
Source: JMIR Diabetes. 2026 Oct 27;11:e88446. doi: 10.2196/88446 (PMC13712406; doi:10.2196/88446)
Supplement: Supplementary file 2 [file diabetes-v11-e88446-s002.docx]

## Multimedia Appendix 2

## Focus Group Discussion Guide

Stakeholders Perspectives on Self-Management and Digital Tools for People with Type 2 Diabetes in Flanders – A Focus Group Study

Interview Guide – The original Dutch Version

| Verwelkoming | |
| --- | --- |
| - Voorstellen van onderzoeksteam | |
| - Introductie/Rationale | Wat is JACARDI + onderzoeksdoelen + funding + projectduur + Pilootproject aan de KU Leuven + onderzoeksdoelen |
|  | Doel van focusgroep discussie |
|  | Introductie van de moderator |
|  | Voorstelling van deelnemers (wie zijn ze + ervaring binnen management van diabetes) |
|  | Interview tips (elkaar laten uitspreken, respectvol blijven, micro uitzetten om onnodige geluid te voorkomen, handopsteken in Teams om bij te dragen aan een bepaald onderwerp, …) |
| - Startvraag – Zelfmanagement definiëren | Wat denken jullie over zelfmanagement in het algemeen? Kan iemand met zijn eigen woorden uitleggen wat zelfmanagement voor u betekent? En wat de belangrijkste pijlers zijn?   - Wat is de bedoeling van zelfmanagement volgens u? - Waarom (zouden we) streven we naar de implementatie van zelfmanagement volgens u? |
|  | Iemand anders nog bedenkingen of aanvullingen? |
| - Vraag 2 – Zelfmanagement in Vlaanderen verbeteren | Wat vindt u van de huidige mogelijkheden tot zelfmanagement in Vlaanderen/België voor mensen met type 2 diabetes mellitus? Leg uit.   - Wat vindt u daar goed aan? - Wat kan beter? |
|  | Iemand anders nog bedenkingen of aanvullingen? |
| - Vraag 3 – Zorgverleners en zelfmanagement | Welke zorgverleners zijn het best geplaatst om patiënten voor zelfmanagement?   - Waarvoor? Met welk doel? - Is er een specifieke taakverdeling nodig? - Zie je een rol voor peer educatie? - Hoe kunnen de artsen, naast diabeteseducatoren, ook aan educatie doen over zelfmanagement voor mensen met T2DM? |
|  | Iemand anders nog bedenkingen of aanvullingen? |
| - Vraag 4 – Factoren die zelfmanagement beïnvloeden | Wat zijn jullie als bevorderende en belemmerende factoren om aan educatie te doen voor patiënten zelfmanagement? Financiering, tijd, training, …   - Bevorderende factoren? - Belemmerende factoren? |
|  | Wat zijn ook bevorderende en belemmerende factoren waarmee patiënten te maken krijgen bij het beoefenen van zelfmanagement?   - Bevorderende factoren? - Belemmeringen? |
|  | Iemand anders nog bedenkingen of aanvullingen? |
| - Vraag 5 – Digitale tools voor zelfmanagement | Wat zou volgens jullie de impact van technologie en nieuwe digitale tools op zelfmanagement en educatie bij mensen met T2DM?   - Hoe zien jullie AI binnen deze context? |
|  | Iemand anders nog bedenkingen of aanvullingen? |
| - Vraag 6 – Bestaande/mogelijke digital tools | Welke digitale middelen zien jullie als nuttig of zelfs nodig om educatie tot zelfmanagement te bevorderen?   - En voor maximale inclusiviteit? Inclusie van digitaal ongeletterde patiënten? En voor equity? - Welke belemmeringen ziet u voor goed gebruik? |
|  | Welke voordelen, kansen, opportuniteiten zijn er en voor welke doelgroep (zorgverleners en patiënten)?   - Probeer dit eens in meer detail uit te leggen? |
|  | Hoe kunnen we deze digitale middelen implementeren om zelfmanagement te bevorderen?   - Wat is zeker mogelijk/haalbaar in Vlaanderen of België? - Welke belemmeringen ziet u voor goed implementatie? |
|  | Hebben jullie ervaring met patiënten portaal (vb. mijngezondheid.be, Helena, NexuzHealth,…). Indien ja, hoe was die ervaring? |
|  | Wat voor andere mogelijkheden/digital tools zijn er voor ziektebeheer? |
|  | Iemand anders nog bedenkingen of aanvullingen? |
| - Vraag 7 – De toekomst in Vlaanderen | Wat voor gevaren zien jullie in die digitale evolutie? |
|  | Iemand anders nog bedenkingen of aanvullingen? |
| Vrije/Bijkomende opmerkingen: Nog bedenkingen of aanvullingen over alle besproken onderwerpen? | |
| - Conclusie/Afsluiting | Iedereen hartelijk bedankten voor hun participatie. |
|  | Men eraan doen denken om de demografische vragenlijst in te vullen en aan ons te bezorgen. |
|  | Als ze een kopie willen van het rapport mogen ze deze ook met ons meedelen |
| Einde van de discussie. | |

Interview Guide – English version (translated by the author)

| Welcome | |
| --- | --- |
| - Introduction of research team | |
| - Introduction/Rationale | What is JACARDI + research objectives + funding + project duration + Pilot project at KU Leuven + pilot objectives |
|  | Objectives of the discussion |
|  | Introduction of the moderator |
|  | Introduction of all participants (who they are + their experience within diabetes management) |
|  | Interview tips (letting each other speak, remaining respectful, turning off the microphone to avoid unnecessary noise, raising your hand in Teams to contribute to a particular topic, etc., ) |
| - Start question – Defining self-management | What do you think about self-management in general? Can someone explain this in their own words? And what the most important pillars are?   - What do you think is the purpose of self-management? - Why do you think we (should) strive to implement self-management? |
|  | Any additional comments or suggestions? |
| - Question 2 – Improving self-management in Flanders | What do you think of the current self-management options in Flanders/Belgium for people with type 2 diabetes mellitus? Please explain.   - What do you like about them? - What could be improved? |
|  | Any additional comments or suggestions? |
| - Question 3 – Healthcare providers and self-management | Which health care providers are best placed for self-management?   - And what are their purpose? - Is a specific division of labor necessary? - Do you see a specific role for peer education? - How can physicians, besides diabetes educators, also educate people with T2DM on self-management? |
|  | Any additional comments or suggestions? |
| - Question 4 – Influencing factors of self-management | What would you consider to be facilitators and barriers to educating on patient self-management? Funding, time, training, etc.   - Facilitators? - Barriers? |
|  | What are also facilitators and barriers that patients face when practicing self-management?   - Facilitators? - Barriers? |
|  | Any additional comments or suggestions? |
| - Question 5 – Digital tools for self-management | What do you think the impact of technology and new digital tools will be on self-management and education for people with T2DM?   - What is your perspective of AI within this context? |
|  | Any additional comments or suggestions? |
| - Question 6 – Existing and potential digital tools | Which digital tools do you consider useful or necessary to promote self-management education?   - And for maximum inclusivity? And for the digitally illiterate patients? And for equity? - What barriers might compromise effective use? |
|  | How can we implement these digital tools to promote self-management?   - What is definitely possible/feasible in Flanders of Belgium? - Which obstacles do you see to effective implementation? |
|  | Do you have experience with patient portals (e.g. mijngezondheid.be, Helena, NexuzHealth, etc.)?  If yes, how was that experience? |
|  | What other options/other digital tools for disease management are there as well? |
|  | Any additional comments or suggestions? |
| - Question 7 – The Future in Flanders | What dangers do you see for the digital evolution? |
|  | Any additional comments or suggestions? |
| Free/additional commentary: Any suggestions or comments on any of the topics discussed? | |
| - Conclusion/Closing Remarks | Thank everyone for their participation. |
|  | Remind participants to complete the demographic questionnaire. |
|  | Mention that participants that may want a copy of the final report may share this with our team. |
| End of the discussion. | |

## Demographic Questionnaire

Stakeholders were invited to complete the following questionnaire to briefly describe the study participants.

2a: Dutch participant demographic questionnaire

| Deelnemer gegevens | |
| --- | --- |
| Naam: |  |
| Leeftijd: |  |
| Wat is uw functie? Patiënt of type zorgverlener? |  |
| Indien zorgverlener, aantal jaren werkzaam in die positie: |  |
| Indien zorgverlener, locatie van werkzaamheid in Vlaanderen (of België): |  |
| Indien zorgverlener, in welke setting werkt u (eerste, tweedelijns of derdelijnszorg)? |  |
| Indien ervaringsdeskundige (lijdend aan diabetes), wat is u diabetesduur? |  |
| Indien ervaringsdeskundige, waar woont u? Stad of platteland? |  |

2b: English participant demographic questionnaire – translated by the author

| Participant information | |
| --- | --- |
| Name: |  |
| Age: |  |
| What is your function? Person with loved experience or healthcare provider? |  |
| For healthcare providers, what is the number of years in that position? |  |
| For healthcare providers, location of employment in Flanders (or Belgium): |  |
| For healthcare providers in the care sector, in what setting do you work (primary, secondary or tertiary/specialized setting)? |  |
| Participants with lived experience, how many years have you been living with diabetes? |  |
| Participants with lived experience, where do you reside? City or countryside? |  |
