# Supplementary material for: Perspectives on Self-Management and Digital Tools for Individuals With Type 2 Diabetes in Flanders: Qualitative Focus Group Study
Source: JMIR Diabetes. 2026 Oct 27;11:e88446. doi: 10.2196/88446 (PMC13712406; doi:10.2196/88446)
Supplement: Supplementary file 3 [file diabetes-v11-e88446-s003.docx]

# Checklist 1

## COREQ Checklist

Reporting of the 32-item Consolidated criteria for reporting qualitative research (COREQ) checklist for interviews and focus groups (1). The page number in the manuscript where each of the items is listed in present in the checklist either with a Yes, No or NA (not applicable).

| **Item No** | | **Guide Questions/Description** | Present in manuscript? |
| --- | --- | --- | --- |
| **Domain 1: Research team and reflexivity** | | | |
| **Personal Characteristics** | | | |
| 1. Interviewer/ facilitator | | Which author/s conducted the interview or focus group? | Yes  Page:11 |
| 2. Credentials | | What were the researcher’s credentials? E.g., PhD, MD | Yes  Page: 11 |
| 3. Occupation | | What was their occupation at the time of the study? | Yes  Page: 11 |
| 4. Gender | | Was the researcher male or female? | NA |
| 5. Experience and training | | What experience or training did the researcher have? | Yes  Page: 11 |
| **Relationship with participants** | | | |
| 6. Relationship established | | Was a relationship established prior to study commencement? | Yes  Page: 5 – 6 |
| 7. Participant knowledge of the interviewer | | What did the participants know about the researcher? e.g. personal goals, reasons for doing the research? | Yes  Page: 5 - 6 |
| 8. Interviewer characteristics | | What characteristics were reported about the interviewer/facilitator? e.g. Bias, assumptions, reasons and interests in the research topic | Yes  Page: 7 |
|  | **Domain 2: study design** | | |
|  | **Theoretical framework** | | |
|  | 9. Methodological orientation and Theory | What methodological orientation was stated to underpin the study? e.g. grounded theory, discourse analysis, ethnography, phenomenology, content analysis | Yes  Page: 6 – 8 |
|  | **Participant selection** | | |
|  | 10. Sampling | How were participants selected? e.g., purposive, convenience, consecutive, snowball | Yes  Page: 5 – 6 |
|  | 11. Method of approach | How were participants approached? e.g., face-to-face, telephone, mail, email | Yes  Page: 6 |
|  | 12. Sample size | How many participants were in the study? | Yes  Page: 9 |
|  | 13. Non-participation Setting | How many people refused to participate or dropped out? Reasons? | Yes  Page: 9 |
|  | 14. Setting of data collection | Where was the data collected? e.g., home, clinic, workplace | Yes  Page: 7 |
|  | 15. Presence of nonparticipants | Was anyone else present besides the participants and researchers? | Yes  Page: 7 |
|  | 16. Description of sample | What are the important characteristics of the sample? e.g. demographic data, date | Yes  Page: 9 |
|  | **Data collection** | | |
|  | 17. Interview guide | Were questions, prompts, and guides provided by the authors? Was it pilot tested? | Yes  Page: 6 |
|  | 18. Repeat interviews | Were repeat interviews carried out? If yes, how many? | NA  Page: 6 – 7 |
|  | 19. Audio/visual recording | Did the research use audio or visual recording to collect the data? | Yes  Page: 7 |
|  | 20. Field notes | Were field notes made during and/or after the interview or focus group? | Yes  Page: 7 |
|  | 21. Duration | What was the duration of the interviews or focus group? | Yes  Page: 7 |
|  | 22. Data saturation | Was data saturation discussed? | Yes  Page: 7 |
|  | 23. Transcripts returned | Were transcripts returned to participants for comment and/or correction? | Yes  Page: 7 |
|  | **Domain 3: analysis and findings** | | |
|  | **Data analysis** | | |
|  | 24. Number of data coders | How many data coders coded the data? | Yes  Page: 7 – 8 |
|  | 25. Description of the coding tree | Did the authors provide a description of the coding tree? | No |
|  | 26. Derivation of themes | Were themes identified in advance or derived from the data? | Yes  Page: 8 |
|  | 27. Software | What software, if applicable, was used to manage the data? | Yes  Page: 7 – 8 |
|  | 28. Participant checking | Did participants provide feedback on the findings? | Yes  Page: 7 |
|  | **Reporting** | | |
|  | 29. Quotations presented | Were participant quotations presented to illustrate the themes/findings? Was each quotation identified? e.g., participant number | Yes  Page: 10 - 16 |
|  | 30. Data and findings consistent | Was there consistency between the data presented and the findings? | Yes |
|  | 31. Clarity of major themes | Were major themes clearly presented in the findings? | Yes  Page: 10 - 16 |
|  | 32. Clarity of minor themes | Is there a description of diverse cases or a discussion of minor themes? | Yes  Page: 10 - 16 |

# References

1.	Tong A, Sainsbury P, Craig J. Consolidated criteria for reporting qualitative research (COREQ): a 32-item checklist for interviews and focus groups. International Journal for Quality in Health Care [Internet]. 2007 Dec 1 [cited 2025 Nov 25];19(6):349–57. Available from: <https://dx.doi.org/10.1093/intqhc/mzm042>
